# Supplementary material for: Early Emergence of 5′ Terminally Deleted Coxsackievirus-B3 RNA Forms Is Associated with Acute and Persistent Infections in Mouse Target Tissues
Source: Vaccines (Basel). 2022 Jul 28;10(8):1203. doi: 10.3390/vaccines10081203 (PMC9413645; doi:10.3390/vaccines10081203)
Supplement: Supplementary file 1 [file vaccines-10-01203-s001.zip › vaccines-1764355-supplementary.pdf]

Supplementary Materials

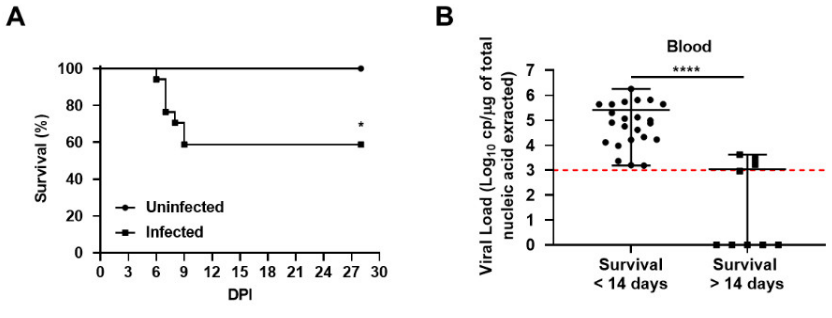

Figure S1. Mortality and viremia in CVB3/28-infected DBA/2J mice. (**A**) Survival curve of infected DBA2/J mice (n = 17; black circle) with 10^6 PFU/ml of CBV3/28 compared to uninfected mice (n = 8; black square). Mock-infected mice had a 100% survival at 28 DPI, whereas infected mice had 41% survival (7/17) (*: $p < 0.05$ by Log-Rank test). (**B**) Comparison of viremia between CVB3/28-infected DBA2/J mice with high or low mortality, to assess a viral load significance threshold of 103 gc/nucleic acid extracted in blood (n = 23: 2–7 DPI; n = 9: 28 DPI). Data represent mean +/− range (****: $p < 0.0001$ by Mann-Whitney U test).

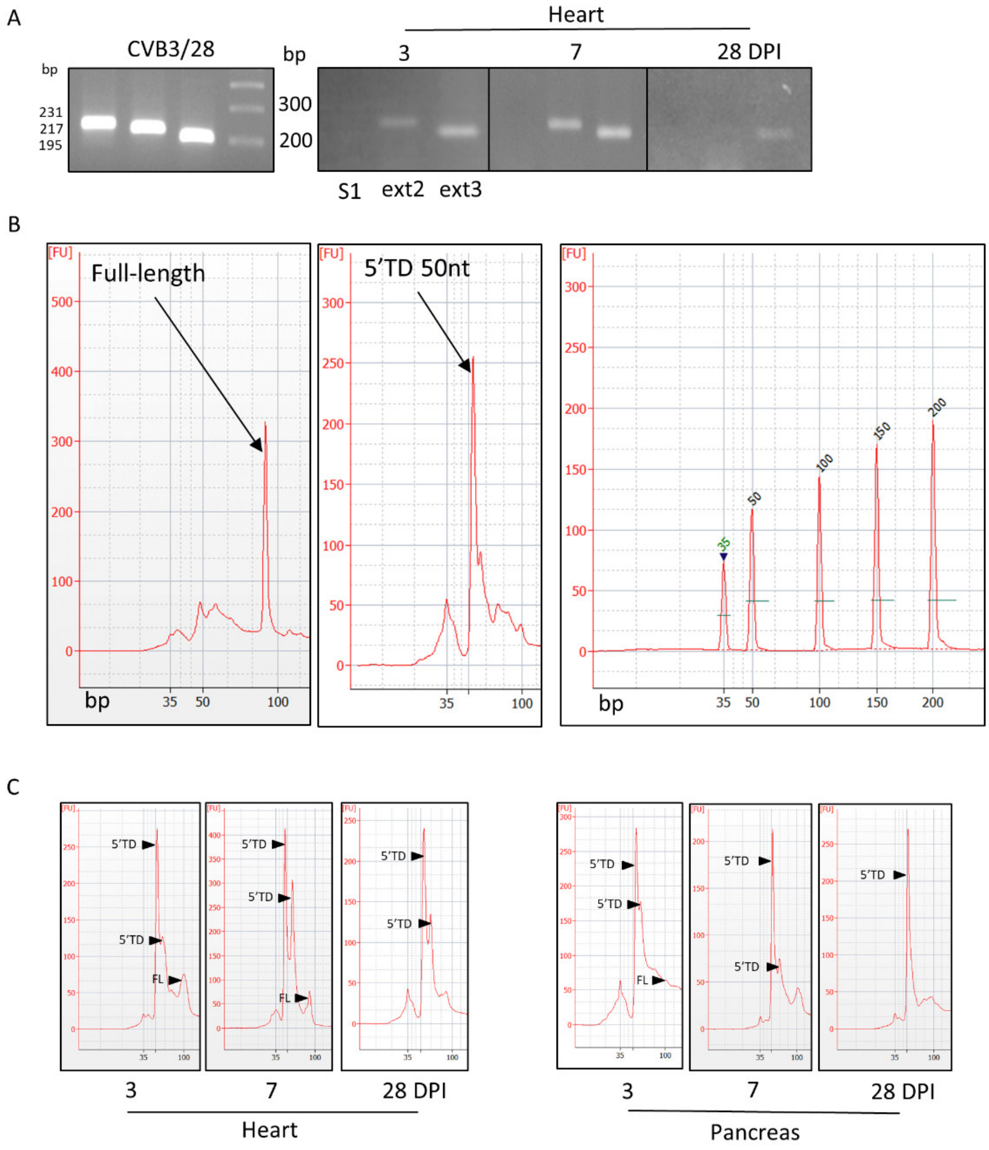

Figure S2. Detection of 5'end deletions in CVB3/28 genome by PCR and micro-electrophoresis. (**A**) PCR was performed with successive priming in the 5'end. S1 binds to full-length forms, ext2 binds to 15 to 34nt of the 5'end, and ext3 binds to 37 to 57nt of the 5'end. Each of the 5' terminus primers amplified cDNA from the wild type CVB3/28 positive control RNA. However, only ext2 and ext3 amplified cDNA from heart samples at 3 and 7 DPI, and only ext3 amplified cDNA from heart samples at 28 DPI. (**B**) Electropherogram following RACE-PCR of synthetic full-length and 5'terminally deleted RNA, and ladder. (**C**) Electropherogram following RACE-PCR of heart and pancreas RNA samples at 3, 7 and 28 DPI. 5'TD : 5'terminally deleted; FL: full-length.
